# Unusual replication dynamics during *Plasmodium falciparum* schizogony

**DOI:** 10.1186/s12936-025-05610-4

**Published:** 2025-11-28

**Authors:** Rosie Berners-lee, Terry K. Smith

**Affiliations:** 1 School of Biology, Biomedical Sciences Research, Complex University of St Andrews North Haugh, St Andrews, KY16 9ST UK; 2 Present Address: Department of Pathology, 10 Tennis Court Road, Cambridge, CB2 1QP UK

**Keywords:** Plasmodium falciparum, Malaria, Schizogony, DNA replication, Replication dynamics, Nuclear divisions

## Abstract

*Plasmodium falciparum* causes the most severe form of malaria in humans, and disease severity is directly linked to parasite proliferation during the erythrocytic cycle. During this cycle, *P. falciparum* replicates via schizogony. This is an unusual form of asexual replication in which the parasite undergoes alternating asynchronous rounds of DNA replication and nuclear division within a shared cytoplasm, followed by a mass cytokinesis event that produces numerous daughter cells. Despite recent advances in high-throughput, single-molecule techniques, clarity on *P. falciparum* replication dynamics remains elusive. These dynamics are likely shaped by its highly AT-rich genome and the unique pressures of schizogony. Clarifying the pressures that shape schizogony and DNA replication may reveal parasite-specific vulnerabilities and inform the development of new antimalarials.

## Highlights

- Schizogony consists of alternating asynchronous rounds of DNA replication and nuclear divisions.
- Replication origins and fork dynamics in *P. falciparum* remain contested.
- Genome composition and limited resources may shape replication during schizogony.

## Background

### The *Plasmodium falciparum* life cycle

**Fig. 1:**
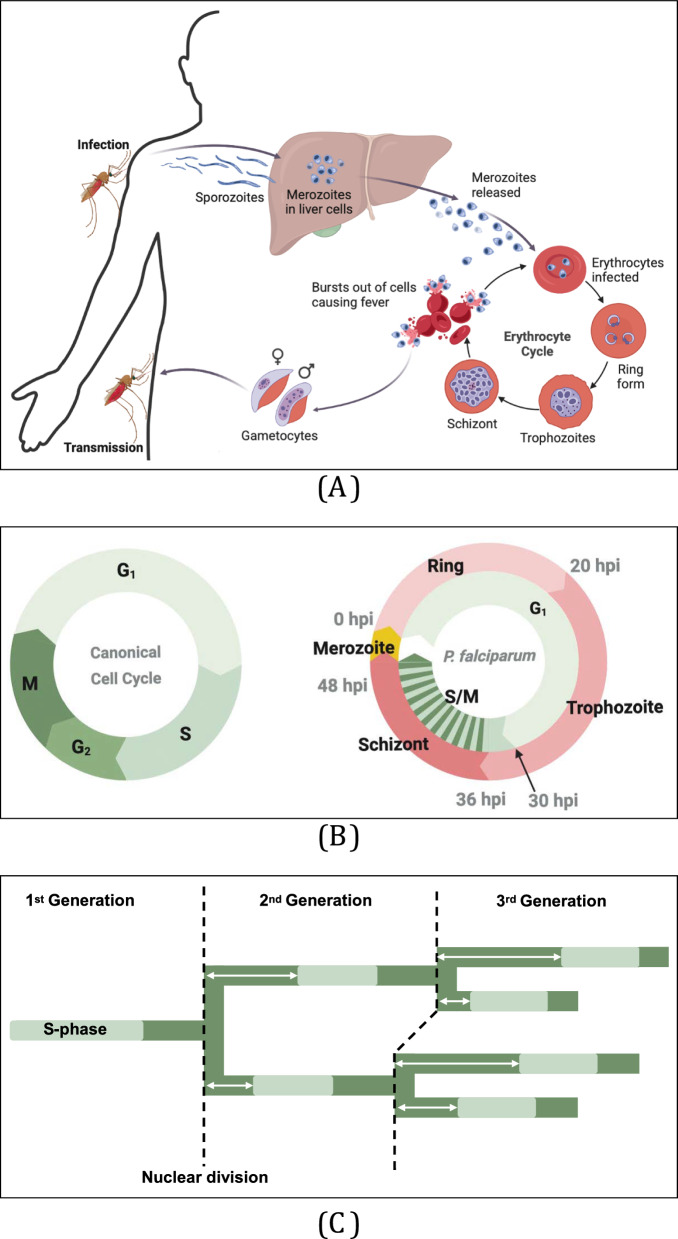
The *Plasmodium falciparum* vertebrate host stages including the erythrocytic cycle. **a** Upon infection via an *Anopheles* mosquito bite, *P. falciparum* sporozoites travel to the liver, where they mature and proliferate before being released into the bloodstream. They invade erythrocytes and undergo the erythrocytic cycle, progressing through the ring and trophozoite stages before producing multinucleated schizonts, which mature into merozoites. Following completion of the schizogony cell cycle, rupture of Erythrocytes releases new merozoites, causing fever and enabling further infection. A subset differentiates into male and female gametocytes, which are transmitted to mosquitoes during a blood meal, continuing the cycle. **b** Comparison of the erythrocytic cycle with the canonical eukaryotic cell cycle. *P. falciparum* matures during the ring and trophozoite stages, which are equivalent to G1. The first S-phase starts towards the end of the trophozoite stage, at around 30 h post invasion (hpi). The Schizont stage then consists of alternating asynchronous rounds of S-phase and mitosis, lacking an intervening G2 phase. **c** Example Nuclear lineage tree showing three consecutive generations of nuclei, based upon Klaus et al*.* [15]. Relative time duration is represented by *horizontal length*. S-phases (*light green*), the intervening time gaps between them (*dark green*), and nuclear divisions (*dashed lines*) are shown. The first S-phase is the longest. Lengths of S-phase and the time between S-phase and nuclear division are similar for sister nuclei, although the latter decreases over successive generations. Asynchrony arises from different time gaps between nuclear division and the start of the next S-phase (*white arrows*). Figures created in BioRender.com

Malaria remains a major global health challenge, with *Plasmodium falciparum* causing the most severe and fatal cases. In 2024, it was responsible for 263 million infections and 597,000 deaths worldwide [1]. *Plasmodium falciparum* has a complex life cycle, alternating between a human host and *Anopheles* mosquito vector (Fig. 1A) [2]. A key feature of the parasite’s replication strategy is schizogony (see Glossary), an unusual form of asexual division where a single parasite undergoes repeated rounds of DNA replication and nuclear division without cytokinesis. This generates a multinucleated syncytium called a schizont, which eventually undergoes a single mass cytokinesis event to produce numerous daughter cells (merozoites) in a single cycle. Schizogony occurs at two points in the life cycle: first during the liver stage and again during the erythrocytic cycle [2].

Infection begins when *P. falciparum* sporozoites are injected into the dermis and actively migrate to blood vessels during a mosquito bite [3, 4]. These travel to the liver, where they replicate via asymptomatic schizogony, producing thousands of merozoites from each parasite. Released into the bloodstream, these merozoites invade erythrocytes and initiate the erythrocytic cycle, progressing through the ring, trophozoite, and schizont stages. Schizogony recurs in the schizont stage, producing 16–32 merozoites per schizont which rupture the erythrocyte and reinvade new ones [2]. This stage is responsible for malaria’s clinical symptoms, causing cyclical bouts of fever (regularly ∼48 h for *Plasmodium vivax* or *Plasmodium malariae*, but irregular for *P. falciparum*) due to synchronized erythrocyte lysis [5]. A subset of merozoites differentiate into gametocytes, which are ingested by feeding female *Anopheles* mosquito during the next blood meal. Once inside the mosquito, the male and female gametes fuse to form zygotes inside the insect’s gut. These zygotes develop into invasive ookinetes, which penetrate and cross the mosquito midgut and forms oocysts. Inside the oocyst, many sporozoites are formed through asexual replication. Sporozoites migrate to the salivary glands, ready to infect a new human host [2].

Malaria severity is directly linked to parasitaemia, which depends upon the number of merozoites produced during each erythrocytic cycle [5], but is also influenced by the sequestration/adherence of infected red blood cells in the microvasculature of the host’s organs. However, schizonts generate variable numbers of merozoites, and the mechanisms regulating this remain poorly understood [6]. Since merozoite number depends upon the number of nuclear divisions and, therefore, successful rounds of DNA replication, understanding how replication is regulated during schizogony may help clarify the pressures which shape the mechanisms governing this variability. *P. falciparum*’s unusually AT-rich genome (80.7%) [7] and apparent prioritization of repeated replication over strict regulatory control leave it inherently replication-stressed, and suggest that its replication dynamics have evolved under distinct selective pressures [8, 9]. These may have driven the emergence of unconventional strategies that differ from human cells, potentially exposing parasite-specific vulnerabilities that could be exploited as antimalarial targets. Recent high-throughput single-molecule techniques (Box 1) now allow genome-wide analysis of DNA replication dynamics in *P. falciparum*, offering new insight at unprecedented resolution [10–12].

## Schizogony dynamics within the erythrocyte cycle

### Comparison with the canonical eukaryotic cell cycle

The conventional eukaryotic cell cycle consists of G1, S-phase, G2, and mitosis, with progression tightly regulated by multiple checkpoints [13]. The *P. falciparum* ring and trophozoite stages correspond to G1, with S-phase beginning late in the trophozoite stage. The subsequent schizont stage involves multiple rounds of S-phase and mitosis (Fig. 1B). However, *P. falciparum* lacks a conventional G2 and many conserved checkpoint mechanisms, giving rise to a distinct replication strategy during schizogony [14]. The *Plasmodium* cell cycle progression is divergent from normal eukaryotes by its lack of canonical cell cycle checkpoint proteins, i.e. p53, ataxia telangiectasia and Rad3-related, ataxia telangiectasia mutated and retinoblastoma protein, additionally, they have an atypical repertoires of cyclins, cyclin-dependent kinases [14].

### Alternating asynchronous rounds of S-phase and mitosis

Schizogony begins ∼30 h post-invasion (hpi) of the erythrocyte (Fig. 1B) [11]. Since. *Pf* PCNA1 dynamically accumulates in actively replicating nuclei, fluorescently tagged *Pf* PCNA1 (*Pf* PCNA1::GFP) has been used as a marker for S-phase activity during schizogony [15]. Using time-lapse live-cell microscopy, Klaus et al*.* [15] tracked S-phase dynamics in schizonts by imaging *Pf* PCNA1::GFP accumulation. This revealed that schizogony consists of alternating asynchronous rounds of S-phase and nuclear division, ruling out alternative models where the genome replicates multiple times before nuclear divisions occur. Each S-phase lasted less than an hour, though the first was significantly longer than subsequent rounds.

A longer initial S-phase was also observed via fluorescently labelled base analogue incorporation into nascent DNA [16]. Fainter staining in later nuclei also suggested slower replication in the final rounds, potentially facilitating synchronization before cytokinesis.

Interestingly, this analysis found a longer delay between karyokinesis and the next S-phase in *P. falciparum* compared to the *Plasmodium knowlesi*, which has a much lower AT content of 60.4% [16]. This may allow time for DNA repair, potentially necessitated by the challenges of replicating an AT-rich genome [8]. However, this would require checkpoint-like regulation, for which there is a lack of evidence.

Nuclear lineage tracking by Klaus et al*.* [15] revealed that intervals between S-phase completion and nuclear division decrease across generations, but both these and S-phase durations remained similar between sister nuclei. In contrast, the time interval between nuclear division and the next S-phase varied between sisters, suggesting this is the primary source of asynchrony (Fig. 1C). S-phase durations were also longer in synchronized sister nuclei compared to asynchronous ones of the same generation, suggesting that competition for limited replication resources and possible nutrient availability slows DNA synthesis when multiple nuclei replicate simultaneously [15 + extra 17].

Competition for essential replication proteins is a well-established regulator of eukaryotic DNA replication dynamics [18]. In *P. falciparum*, this limitation is likely exacerbated during schizogony, where many replicating nuclei share the same cytoplasm. Arrest of replication in some nuclei, observed by McDonald and Merrick [16], was also attributed to limited replication factor availability and may represent an additional source of asynchrony. Together, these findings suggest that *P. falciparum* may have evolved asynchronous replication as a strategy to reduce competition for replication factors and / or nutrient availability, enabling more efficient DNA synthesis and maximizing merozoite production [15 + 17].

### Determining the number of merozoites produced

Merozoite production varies widely between schizonts. While the number of merozoites produced directly influences disease severity, the factors that control this output, and the reasons for its variability, remain poorly understood (Box 2) [6]. Variation in merozoite number may partly arise from replication arrest in individual nuclei, since early arrests can eliminate an entire nuclear lineages, massively reducing merozoite production [16]. This suggests that within-schizont differences in nuclear fate may contribute to variation between schizonts, potentially influenced by pressures on DNA replication dynamics. Two more deterministic models have also been proposed: a timer model where schizogony proceeds for a fixed duration; and a counter model where it ends after a set number of nuclear divisions [6]. There is no direct evidence supporting the timer model. However, *P. knowlesi*, which has a much shorter erythrocytic cycle of ∼24 h (compared to *P. falciparum*’s ∼48 h), also produces fewer merozoites per schizont [16]. This suggests that the duration of schizogony may influence merozoite output, although a causal relationship remains unproven. While evidence for the counter model is also limited, it is supported by mathematical modelling, which demonstrates that longer S-phase durations are not associated with reduced merozoite production, as would be expected under a timer model [13].

The mechanism underlying this counter remains unclear. Variation in merozoite production implies the counter’s cap differs between schizonts. One possibility is that this limit is genetically determined. If so, merozoites would produce similar merozoite numbers to their parents in subsequent cycles [18]. Such variation could allow adaptation to extracellular conditions such as nutrient availability in the bloodstream, which is known to influence schizont output [19]. For instance, *P. falciparum* grows better in diabetic patients with high glucose levels [20], but produces fewer merozoites under nutrient-restricted conditions in mice [21]. Stürmer et al*.* [17] suggest that malaria proliferation responds to the levels of extracellular resources allowing modification of progeny number. Thus, understanding both intra- and extracellular factors influencing merozoite production will provide valuable insights for treatment development and disease management. An important intracellular determinant is the success of DNA replication. Although DNA replication dynamics may not dictate how the number of nuclear divisions is determined, successful DNA replication is crucial for enabling their completion. Understanding how replication proceeds in *P. falciparum*, and how this differs from canonical models is, therefore, central to understanding the pressures and regulation shaping schizogony.

## DNA replication during *P. falciparum* schizogony

### Canonical eukaryotic DNA replication

**Fig. 2:**
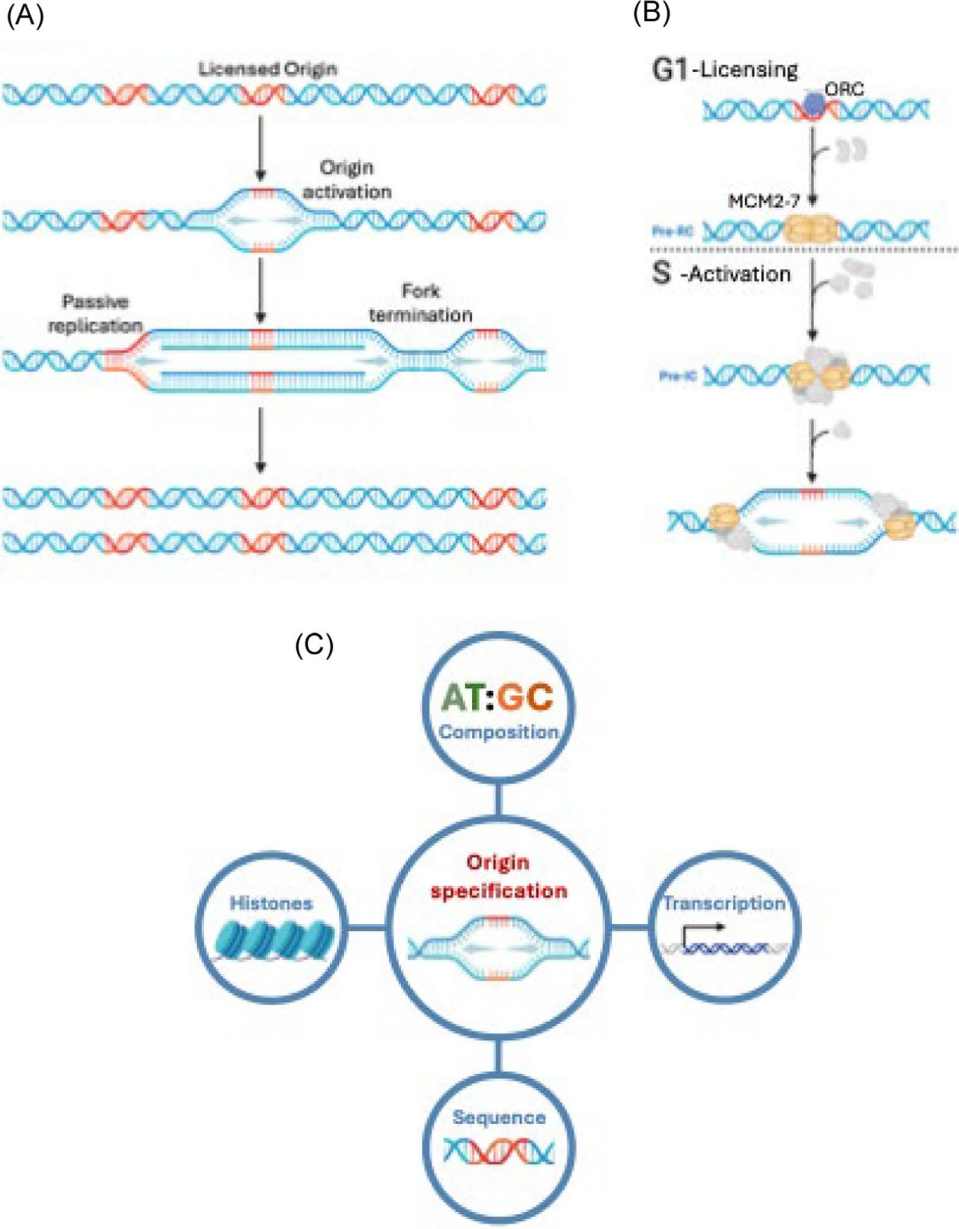
Features of DNA replication. **a** Overview of eukaryotic DNA replication dynamics. Licensed origins (*red*) are *activated* throughout S-phase, initiating bidirectional DNA replication. Not all licenced origins are activated. Instead, some are *passively replicated* by oncoming replication forks. Replication forks *terminate* upon either converge or reaching the ends of chromosomes. **b** Mechanism of initiating DNA replication, conserved across most eukaryotes. Initiation is split into two stages, licencing and activation. Licencing starts by ORC (*purple*) binding to origins which, together with additional replication factors Cdt1 and CDD6 (*grey*) transiently bind, facilitates the loading of two MCM-2-7 helicases (*light beige*) to form the pre-replication complex (pre-RC). Other replication factors Sld2, Sld3, Dpb1 and subsequently Mcm10 (*grey*) transiently bind that facilitate origin activation. Orthologues of both of both ORC and MCM2-7 subunits have been identified in *P. falciparum*. **c** Features proposed to influence origin licensing and activation: AT:GC base composition, specific sequences, histone modifications, and transcriptional activity. Figures created with BioRender.com

Canonical eukaryotic DNA replication initiates at multiple sites, known as origins, which are activated stochastically at different times throughout S-phase [22]. During G1, potential origins are licensed by origin recognition complex (ORC) binding to DNA which, together with additional factors, loads two MCM2-7 helicases around duplex DNA to form a prereplication complex, marking the origin as replication-competent [22]. A subset of licensed origins are activated in S-phase, initiating bidirectional DNA synthesis from two replication forks that continue until they merge with other forks or reach chromosome ends (Figs. 2A–2B) [23]. Origin specification varies across eukaryotes and is influenced by multiple features including sequence composition, chromatin state, DNA secondary structures, and transcriptional activity (Fig. 2C) [24]. Understanding what determines *P. falciparum* origin locations during schizogony is crucial for uncovering how DNA replication is regulated and how it may have adapted to its highly AT-rich genome and unique mode of replication (Box 2) [10].

### Sites of origin licencing

To map potential *P. falciparum* origins genome-wide, Totanes et al*.* [10] and Castellano et al*.* [11] used ChIP-seq to identify where ORC subunits associate with DNA. Totanes et al*.* [10] reported a high density of *Pf* ORC1 binding sites, approximately one per kilobase (kb). This could provide excess dormant origins to serve as backups if replication forks are disrupted. While this safeguard is common across eukaryotes, it may be especially important in *P. falciparum*, given its elevated replication stress, because of having to produce multinucleated schizonts [8, 10]. In contrast, Castellano et al*.* [11], who mapped both *Pf* ORC1 and *Pf* ORC2, but only considered overlapping sites as potential origins, identified five times fewer binding sites, which were often clustered. This discrepancy may reflect differences in experimental techniques or ChIP-seq peak calling thresholds [11]. ChIP-seq was also used to identify genome-wide binding sites of the helicase subunit *Pf* MCM6 [25], revealing similar distribution to *Pf* ORC1 sites reported by Totanes et al*.*

[10]. However, the broader peaks suggested that MCM2-7 may translocate along DNA before.

origin activation, a mechanism gaining support across eukaryotes [26].

In most eukaryotes, origins are not sequence-specific; although *Saccharomyces cerevisiae* is an exception, with origins occurring at defined autonomously replicating sequences (ARS) [27]. The sequence specificity of *P. falciparum* origins remains unresolved. A bioinformatic autocorrelation analysis identified numerous *Pf* ARS sequences sharing features with *S. cerevisiae* ARSs. These were enriched for nascent DNA and increased plasmid stability, suggesting they may act as origins [28]. Supporting this, ChIP-qPCR showed preferential *Pf* MCM6 binding at selected *Pf* ARS compared to non-ARS controls [25]. Although ChIPseq revealed that *Pf* MCM6 binding sites rarely overlapped precisely with *Pf* ARS sequences, most were within 5 kb, consistent with a model where MCM2-7 translocates prior to origin activation [25]. However, neither Totanes et al*.* [10] nor Castellano et al*.* [11] reported sequence specificity in *Pf* ORC subunit binding. This suggests that *Pf* ARS may not be essential origin features, but instead increase the likelihood of sites functioning as origins. While not central to determining *P. falciparum* replication dynamics, *Pf* ARSs may still have practical value, for example, in designing more stable plasmids [25].

Despite the preference for AT-rich origins in most eukaryotes [24], both Totanes et al*.* [10] and Castellano et al*.* [11] found *Pf* ORC binding sites to be enriched at relatively GC-rich sequences. However, given the extremely high AT content of the *P. falciparum* genome, the absolute GC content of *Pf* ORC binding sites is actually comparable to origins in many other eukaryotes. This suggests that a certain GC-content range may be a conserved feature of eukaryotic origins and it has even been proposed that GC-content alone could be sufficient to determine *Pf* ORC binding [10]. *Pf* ORC binding was also enriched at G4FS (G-quadruplex Forming Sequences- DNA secondary structures consisting of stacked guanine tetrads) [10, 11], which have been associated with metazoan origins [24]. However, it remains unclear whether this is causal, or simply reflects the higher GC content of *Pf* ORC binding sites.

Castellano et al*.* [11] reported enriched *Pf* ORC binding within coding regions, particularly in highly transcribed genes. Similarly, *Pf* MCM6 binding was enriched within coding regions [25]. However, Totanes et al*.* [10] found no correlation between *Pf* ORC1 binding and coding regions, possibly due to the much higher number of *Pf* ORC1 sites identified in their study. Totanes et al*.* [10] also reported a negative correlation between *Pf* ORC1 binding and gene expression, directly opposing findings by Castellano et al*.* [11]. Origins in highly transcribed regions may be more accessible to replication proteins [29], while those in lowly transcribed regions avoid replication-transcription conflicts, a major source of replication stress [30]. Clarifying the relationship between transcriptional activity and *P. falciparum* origin preference will provide important insights into the hierarchy of selective pressures shaping its replication strategy.

In *S. cerevisiae*, histones H3 and H4 are dynamically regulated around origins [31]. In *P. falciparum*, the histone acetyltransferase *Pf* MYST preferentially acetylates H4K8 at *Pf* ARS sites in a DNA replication-dependent manner, suggesting similar histone involvement [32]. Supporting this, liquid chromatography-tandem mass spectrometry identified an association between *Pf* MCM6, H4 and H3, and comparative ChIP-seq analysis across multiple studies found substantial overlap between *Pf* ORC1, *Pf* MCM6 and H4K8Ac sites [25]. However, Totanes et al*.* [10] and Castellano et al*.* [11] reported no correlation between histone modifications and *Pf* ORC binding, leaving role of histones in *P. falciparum* origin licensing unclear. Together, these findings suggest that, like other eukaryotes, origin specification in *P. falciparum* results from a complex interplay of genomic, transcriptional, and chromatin features. However, the relative contribution of each remains unresolved.

### Sites of origin activation

The stochasticity of origin activation means that the subset of origins activated and their activation timings vary between replicative cycles (Fig. 2A) [33]. Therefore, population averaging techniques such as ChIP-seq are unsuitable for identifying activation sites, as they smooth over heterogeneity between cycles. To overcome this, Castellano et al*.* [11] and Totanes et al. [10] investigated sites of origin activation and replication fork speeds using the recently developed single-molecule techniques NanoForkSpeed (NFS) and DNAScent, respectively (Box 1) [34, 35]. Neither study found direct overlap between origin activation sites and *Pf* ORC binding, though this was somewhat expected due to technical differences. Nonetheless, each study reported that activation site characteristics aligned with their respective findings on *Pf* ORC binding sites. Consequently, both studies found activation sites were GC-rich and enriched in coding sequences, but reported opposite correlations with transcription and no correlatiosn with histone modifications. Notably, DNAscent analysis of origin activation in *P. knowlesi*, which has a more balanced AT:GC composition, showed no preference for GC-rich sequences [9], providing further evidence that *P. falciparum* origin selection is shaped by its AT-rich genome

#### Replication fork speeds

Castellano et al*.* [11] reported an average replication fork speed of 1.6 kb/min, close to the 1.2 kb/min previously measured by the gold-standard DNA combing technique [36], and comparable to rates observed in humans and *S. cerevisiae*. In contrast, Totanes et al*.* [10] reported a much lower average speed of 0.6 kb/min. Their estimate was supported by a simulation-based model of *P. falciparum* chromosome 1, which showed that their measured fork speed and origin distribution were consistent with completing S-phase within the expected timeframe [10]. Fork speed is generally calculated by dividing the length of labelled nascent DNA by the duration of the labelling pulse. Therefore, the lower estimate from Totanes et al*.* [10] may reflect their use of a much longer pulse than Castellano et al*.* [11], potentially masking transient fork stalling by averaging over stalled and active forks. Fork stalling may be particularly prevalent in *P. falciparum* due to its high AT-richness and lack of conserved regulatory mechanisms [35], highlighting the need to investigate the extent and impact of fork stalling in *P. falciparum*.

Supporting this, DNAScent analysis found that replication forks in the less AT-rich *P. knowlesi* were ∼50% faster than in *P. falciparum* [9]. Furthermore, *P. knowlesi* forks slowed down in AT-rich regions whereas *P. falciparum* forks did not, suggesting that *P. falciparum* may have evolved to tolerate its AT-rich genome [9]. Alternatively, slower forks or stalling in *P. falciparum* may reflect competition for limited replication resources, a well-established phenomenon across eukaryotes [37], likely exacerbated by multiple simultaneous rounds of DNA replication. Consistent with this, *P. knowlesi* schizonts produce considerably fewer merozoites, suggesting this pressure is weaker [16]. Comparative studies across species with biased nucleotide compositions could help disentangle the effects of replication burden and genome architecture on replication dynamics.

## Concluding remarks

Current understanding of DNA replication and nuclear division dynamics during *P. falciparum* schizogony remains limited and, at times, contradictory. We propose that *P. falciparum*’s replication programme is shaped by the combined pressures of its highly AT rich genome, lack of canonical checkpoints, and the strain of executing multiple rounds of DNA replication within a shared cytoplasm. These pressures likely influence not only the molecular-scale replication dynamics, such as origin usage and fork speeds, but also higher level organizational features, including asynchronous nuclear divisions and variable merozoite output. For example, competition for limited replication factors and the challenges of replicating an AT-rich genome may contribute to slower fork speeds and increased stalling at the molecular level. These pressures could also drive selection for asynchronous nuclear divisions as a strategy to minimize competition between nuclei, and may contribute to variability in merozoite output by promoting replication arrest in some nuclei due to global replication stress. Recent advances in high-throughput single-molecule techniques are accelerating our ability to map replication dynamics genome-wide at unprecedented resolution [31, 32]. Applying these techniques across species and conditions will be central to understanding how replication programs evolve under distinct selective pressures and may clarify how *P. falciparum* has adapted to replicate under extreme genomic and cellular constraints (Box 2). Linking replication dynamics to merozoite production could offer new insight into how this shapes parasite fitness and disease severity, ultimately uncovering how *P. falciparum* balances conflicting pressures during schizogony and potentially exposing new targets for antimalarial therapies.

## Box.1 Uncovering replication dynamics via sequencing with base analogues


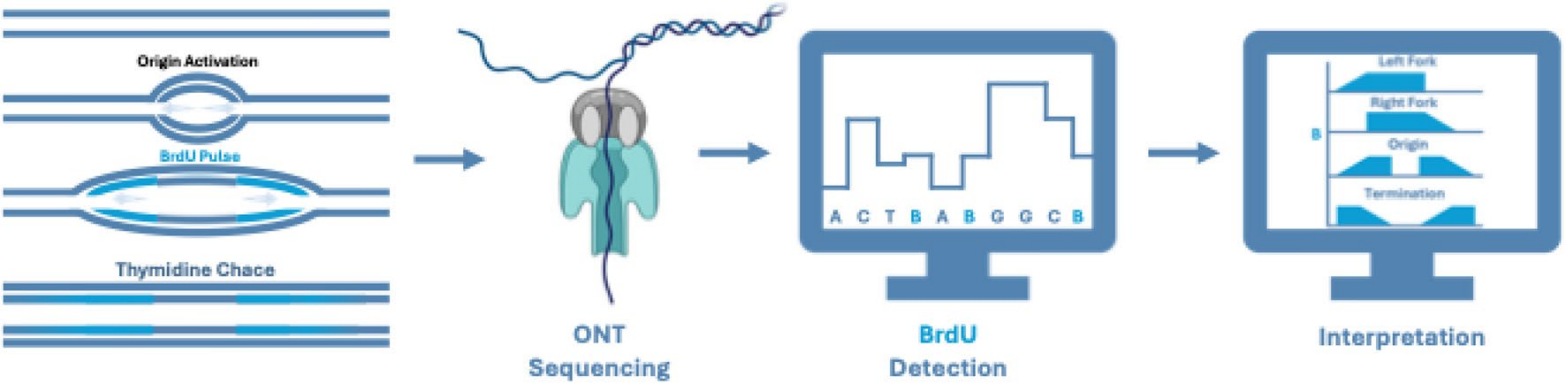
 Inferring replication fork dynamics from BrdU incorporation: Nascent DNA is labelled with BrdU (*light blue*) during a pulse, followed by a thymidine chase. ONT sequencing detects changes in electrical current as DNA passes through the nanopore, with different bases causing distinct shifts. Software such as DNAScent and NFS can then identify BrdU incorporation by recognising subtle shifts in these currents. Patterns in BrdU incorporation are then used to infer key features of DNA replication dynamics. Figure created in BioRender.com.

High-throughput, genome-wide, single-molecule approaches are invaluable for studying DNA replication dynamics. Advances in computational analysis of ONT sequencing data have enabled the detection of base analogues incorporated into nascent DNA, allowing replication dynamics to be mapped genome-wide. ONT sequencing works by unwinding DNA and passing it through a nanopore embedded in a charged membrane. Each nucleotide produces a characteristic current shift, enabling base identification. Base analogues, such as BrdU, cause subtler current shifts, which recent software developments can now detect [31, 32]. Pulsing replicating cells with BrdU, followed by a thymidine chase, labels newly synthesized DNA, which can then be mapped back onto the genome. The resulting BrdU incorporation patterns reveal replication fork movement and provide insights into replication dynamics (Box 1 Figure). Replication fork directionality (RFD) is inferred by identifying regions of baseline, above-baseline, increasing, and decreasing BrdU incorporation. From RFD, replication initiation and termination sites, as well as fork speeds, can be determined at a single-molecule level. Long-read sequencing advances increase the information per read. This is crucial because, while these techniques are single-molecule, they are not single-nucleus, meaning only replication dynamics within the same read are definitively from the same S-phase.

Box. 2 Outstanding Questions What regulates the number of merozoites produced per schizont, and how does replication success contribute to this variability?

What are the molecular mechanisms that coordinate asynchronous S-phases and nuclear divisions during schizogony?

How does the extreme AT-richness of the *P. falciparum* genome influence origin usage and fork progression?

To what extent does competition for shared cytoplasmic resources influence replication dynamics and coordination of nuclear divisions during schizogony?

What determines the sites of origin licensing and activation in *P. falciparum*?

How frequent is replication fork stalling during *P. falciparum* DNA replication and what are the impacts of this?

## Data Availability

No datasets were generated or analysed during the current study.

## Glossary

Autonomously replicating Sequence (ARS): Sequence specific origins in S. cerevisiae.
Bromo-deoxyuridine (BrdU): A thymidine nucleoside analogue, often used for labelling nascent DNA. BrdU differs from thymidine by having a bromine atom in place of thymidine’s CH3 group.
Chromatin immunoprecipitation followed by Sequencing (ChIP-seq): A high-throughput technique for mapping genome-wide protein-DNA interactions. DNA-bound proteins are cross-linked, the chromatin is fragmented, and unbound DNA is removed. The protein of interest is immunoprecipitated, and the associated DNA is sequenced and mapped to a reference genome to identify binding sites.
G-quadruplex forming gequences (G4FS): G-rich regions which form DNA secondary structures consisting of stacked guanine tetrads, where four guanine bases associate through hydrogen bonding, stabilized by monovalent cations such as K+ or Na+.
H4K8Ac: Acetylation of histone H4 at lys 8, associated with opening chromatin during DNA replication.
Mini-chromosome maintenance 2-7 (MCM2-7): Highly conserved eukaryotic replicative helicase, which are loaded onto DNA during origin licensing and unwinds double stranded DNA during replication.
Oxford nanopore technologies (ONT) sequencing: Heterohexameric protein complex that binds to origins and initiates origin licensing by recruiting additional replication factors.
P. falciparum proliferating nuclear antigen 1 (Pf PCNA1): P. falciparum orthologue of PCNA, a DNA polymerase cofactor that acts as a sliding clamp and binding platform for multiple replication fork components.
Schizogony: Multiple rounds of DNA replication and nuclear division within a single cytoplasm, without cytokinesis.
